# Nutcracker Syndrome in a 77-Year-Old Female With Bilateral May-Thurner Syndrome: A Case Report

**DOI:** 10.7759/cureus.43996

**Published:** 2023-08-23

**Authors:** Shujee Yawar, Rabail Saeed Shaikh, M. Akram Khan

**Affiliations:** 1 Cardiology, Cardiac Center of Texas, McKinney, USA

**Keywords:** left renal vein entrapment:, bilateral, pelvic congestion syndrome, case reports, nutcracker syndrome, may-thurner syndrome

## Abstract

Nutcracker syndrome (NCS) is an uncommon condition that predominantly affects the left renal vein (LRV) because of its entrapment between the aorta and superior mesenteric vein. It can result in pain in the left flank or back, hematuria, and proteinuria. May-Thurner syndrome (MTS) is described as a condition in which the left common iliac vein is compressed by the right common iliac artery, causing swelling, fullness, venous ulcers, or varicose veins in the leg. We present a case of a 77-year-old female who had these symptoms for over a decade, until she was diagnosed in 2017. Initially, she experienced swelling and pain in her left leg, which began in 2005; however, treatment did not begin until she had her left ovarian vein embolized in 2017. Her symptoms returned the following year, leading to the diagnosis of bilateral MTS. Owing to recurring symptoms in 2022, a repeat venogram revealed bilateral external iliac vein constriction, requiring intervention. She presented to our clinic in 2023 after being referred by her cardiologist because of persistent back pain and venous congestion. This led to the findings and diagnosis of NCS with bilateral MTS.

## Introduction

Nutcracker syndrome (NCS) is the entrapment of the left renal vein (LRV) between the aorta posteriorly and superior mesenteric artery anteriorly. An aortomesenteric angle of <35°-39 °is considered diagnostic; however, it remains a diagnosis of exclusion and requires extensive imaging. The exact prevalence is currently unknown owing to a lack of diagnostic criteria [1]. It was previously believed to be more prevalent in females, but the latest studies showed equal prevalence in both sexes. It can be found at any age, from childhood to the elderly, but the peak prevalence is in the second to third decades and in young adults [2]. It is associated with May-Thurner syndrome (MTS) {3}, which is the compression of the common iliac vein by the common iliac artery, typically on the left side. In our case, there was a rare incident of MTS occurring bilaterally, which led to the subsequent finding of NCS.

## Case presentation

A 77-year-old woman presented with intermittent sharp pain in the epigastric and left flank regions. She had a history of bilateral MTS with stent placement and pelvic congestion syndrome with left ovarian vein embolization.

She first complained of left leg swelling and calf pain in 2005; however, a venogram performed at that time showed only mild narrowing of 20% in the superficial femoral artery. It was concluded that the symptoms were not vascular in origin. Her next documented reports were from February 2017, when she presented with a prolonged history of pelvic pain and lower extremity swelling, which worsened while standing. All risk factors for thromboembolism, i.e., immobilization, prolonged rest, hereditary thrombophilia, and malignancy, were ruled out after a thorough patient interview. A venogram performed in March 2017 showed significant stenosis of the left external iliac vein of approximately 70-80% and was treated with balloon angioplasty, dilating the vein to 10 mm. Venogram of the LRV also demonstrated significant reflux into an enlarged left ovarian vein, confirming the diagnosis of pelvic congestion syndrome. Follow-up was scheduled to embolize the left ovarian vein. In April 2017, venography of the left ovarian vein was performed, followed by its embolization through a right internal jugular venous access. Seven Terumo detachable platinum coils ranging from 10 to 14 mm were deployed along the left ovarian vein.

In August 2018, owing to the recurrence and worsening of symptoms despite prior intervention, the patient obtained a second opinion from a vascular specialist. Venous duplex ultrasound was performed, and she was diagnosed with class III varicose veins according to the Clinical, Etiology, Anatomic, Pathophysiology (CEAP) classification of venous disorders. A conservative therapy trial with medical grade stockings failed to relieve her symptoms, so the patient wished to proceed with surgical therapy and was counselled regarding radiofrequency ablation (RFA) for the left great saphenous vein, right great saphenous vein, and right small saphenous vein. It was successfully performed in 2019 without any complications.

Two months later, in October 2018, she followed up with a doctor with persistent symptoms of lower extremity swelling and pain. A venogram of the bilateral femoral, common iliac, and external iliac veins was obtained using intravascular ultrasound (IVUS). Reports showed significant stenosis of 70-75% of the right common iliac vein and severe left common iliac vein stenosis of >80%, consistent with the diagnosis of bilateral MTS. Intervention was performed via repeat venography a month later in November 2018 with placement of wall stents measuring 16 mm × 90 mm and 18 mm × 90 mm in the right and left common iliac veins, respectively. A post-stenting venogram showed restoration of the vessel lumen and flow.

**Figure 1:**
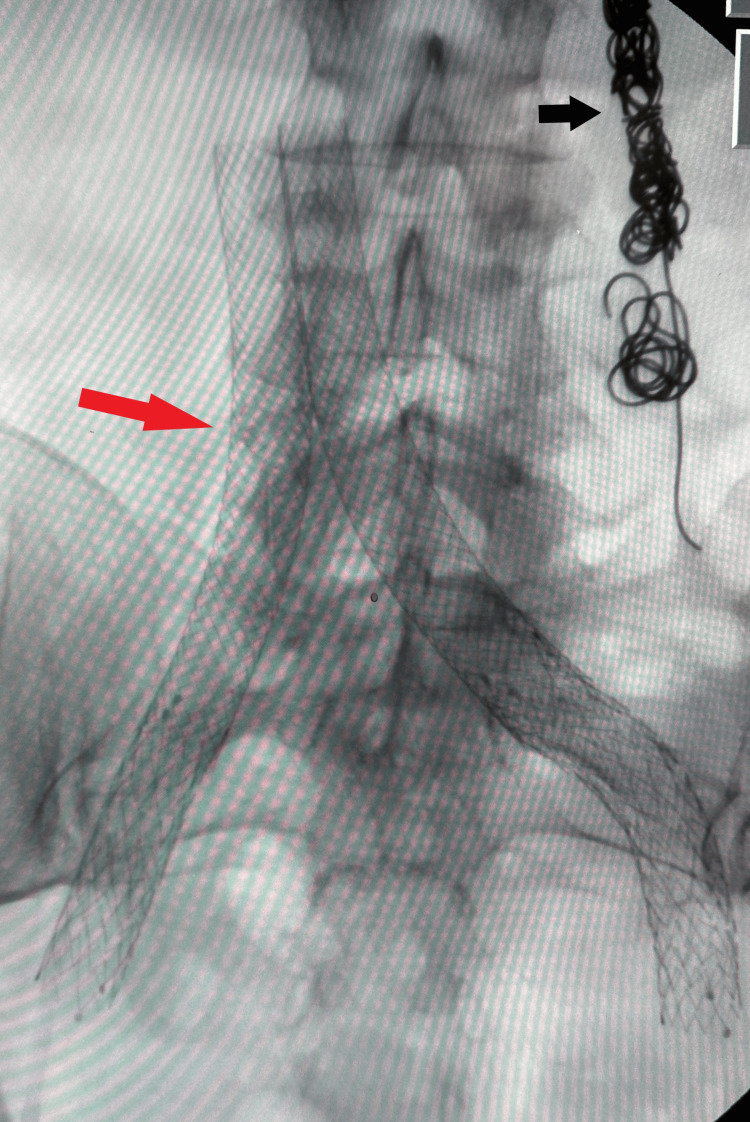
CT image showing stents in the bilateral internal and external iliac veins (red arrow) and coils in the left ovarian vein (black arrow).

In May 2022, after re-emergence of her symptoms of pelvic pain and lower extremity swelling, she underwent evaluation for stent patency by venography with IVUS. The procedure was remarkable for anterior-posterior compression of the bilateral external iliac veins proximal to the previous stents placed in the common iliac veins. Anterior-posterior compression of the LRV was also noted. However, it was decided to perform the intervention solely in the external iliac veins with the placement of venous stents measuring 14 mm × 60 mm and 14 mm × 80 mm in the right and left external iliac veins, respectively (Figure 1).

**Figure 2:**
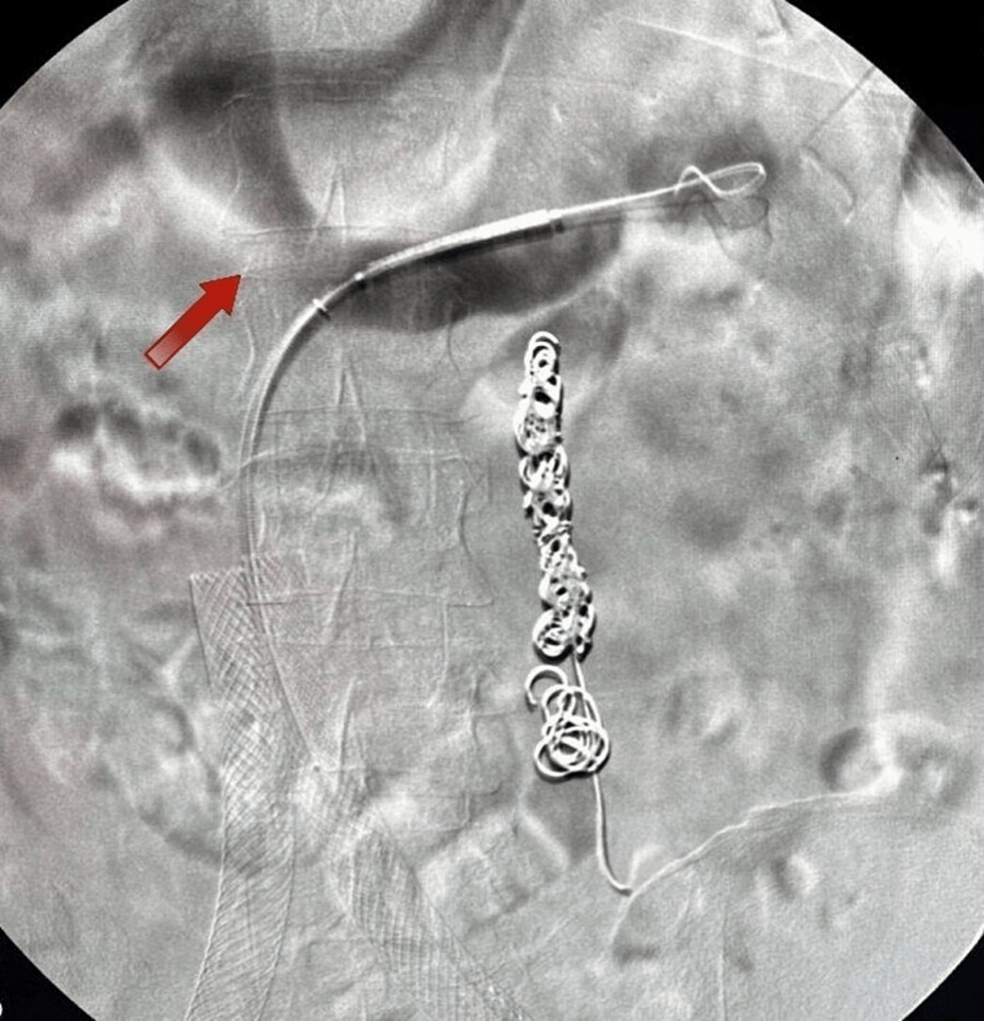
Venogram showing the constriction of the left renal vein (red arrow).

**Figure 3:**
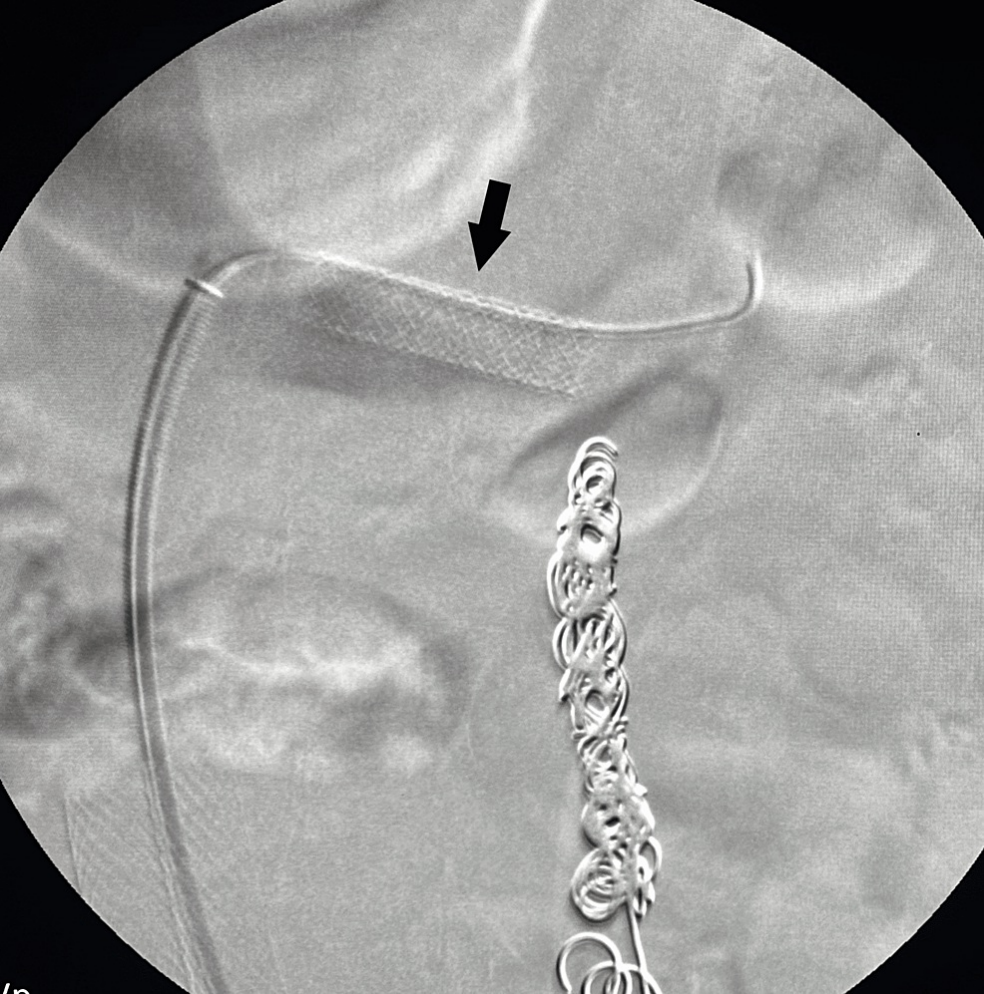
Placement of a 9 mm x 37 mm x 75 mm stent in the left renal vein (black arrow).

**Figure 4:**
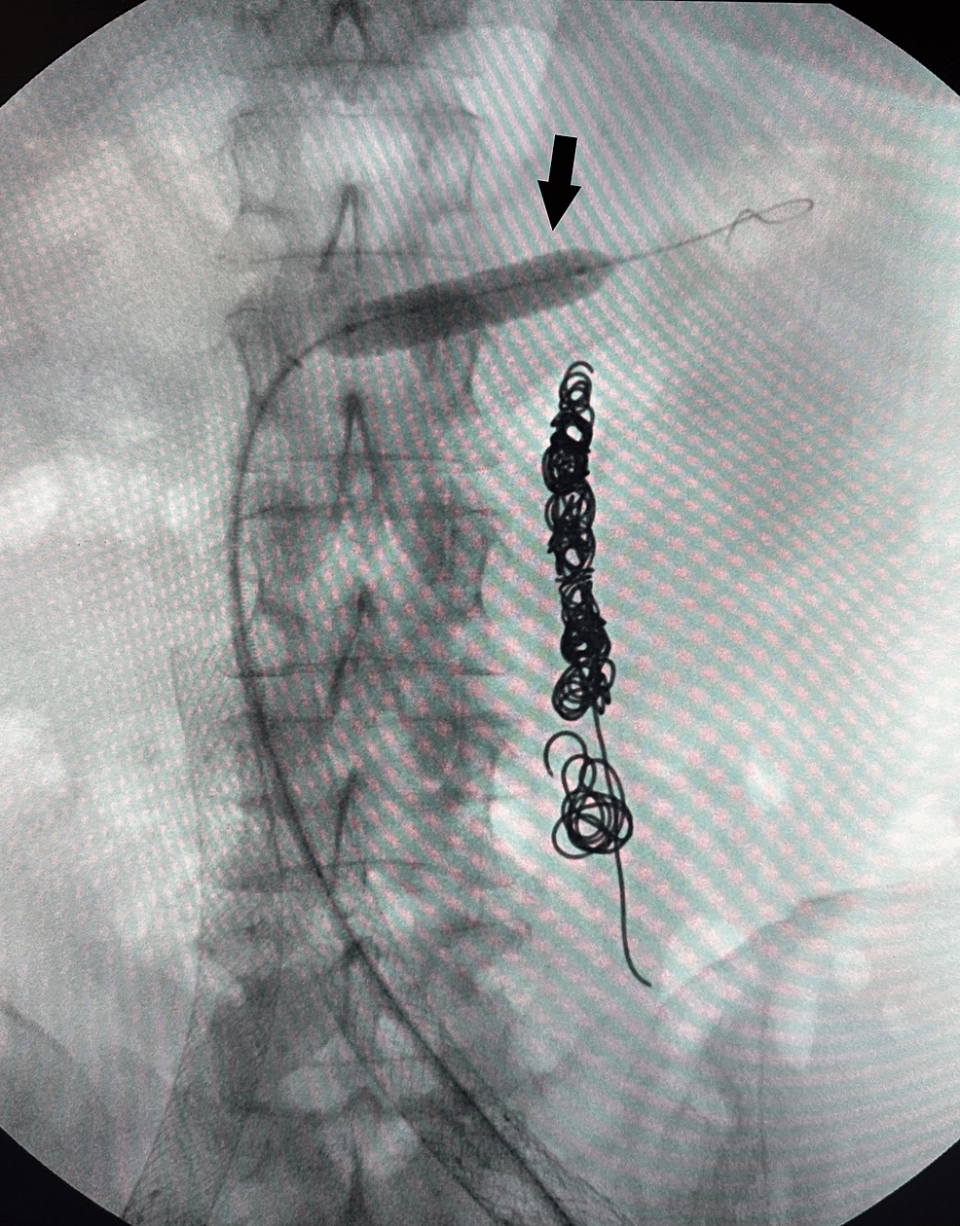
Left renal vein stent post dilated with a balloon to 10 mm (black arrow).

In March 2023, she underwent a small left saphenous vein RFA due to a significant reflux of 860 ms. A month later, she presented with symptoms of flank and epigastric pain, which combined with her previous report of LRV compression, leading to the diagnosis of NCS. An LRV venogram with IVUS was performed in May 2023 to confirm the diagnosis (Figure 2). Due to the significant compression seen on IVUS, an intervention was done, and a stent measuring 9 mm × 37 mm × 75 mm was inserted and post-dilated to 10 mm with a balloon (Figures 3, 4). After renal vein stent placement, her symptoms completely resolved. Follow-up with LRV ultrasonography was scheduled in September 2023 to confirm stent patency.

## Discussion

NCS is a diagnostic challenge due to the rarity of the disease and its varying manifestations, including the possibility of bilateral MTS. The lack of set diagnostic criteria means that even patients with typical symptoms, as in our case, require a long time and extensive testing before diagnosis, which results in prolonged morbidity. This emphasizes the need for a diagnostic criteria for early identification of patients with venous congestion symptoms.

The NCS is defined by the engorgement of the aortomesenteric region of the LRV, which presents with microhematuria, proteinuria, and flank pain. When the LRV is compressed, blood from the kidneys flows into veins with a lower resistance, such as the left gonadal vein. This leads to a long cascade of events, from pelvic congestion syndrome to MTS to right iliac vein congestion.

MTS was coined by Dr. May and Dr. Thurner in 1957 to describe the cause of iliac vein obstruction. Compression of the left iliac vein is caused by continuous pulsation of the overlying right common iliac artery, which results in the loss of elasticity and fibrotic changes in the vessel wall, causing stenosis and presenting with symptoms of venous hypertension. It is classically only present in the left common iliac vein because of compression by the right common iliac artery [3]. Only two cases of bilateral MTS have been reported in so far, one in 2018 due to aneurysmal enlargement of the common iliac veins and the second in 2023 due to thrombus from the left common iliac vein causing obstruction in the right common iliac vein [4,5]. Right-sided MTS has only been reported in one patient who had a left-sided inferior vena cava [6].

Our case is unique in the sense that it is the first case of NCS associated with bilateral MTS, which is due to the increased stress from high blood flow leading to vessel stenosis without any obvious signs of aneurysm or thrombosis.

Current diagnostic practices include Doppler ultrasonography, MRI, CT, phlebography, and IVUS [2]. However, owing to the absence of established criteria, it is still a challenging diagnosis based solely on imaging. Therefore, clinical presentation is of utmost importance. Doppler ultrasound (DUS) of the LRV to measure peak velocity remains the initial method of choice. It has high specificity and sensitivity of 89-100% and 69-90% respectively [2]. If DUS is inconclusive, CT or MRI is used to measure the angle between the aorta and superior mesenteric artery and the LRV diameter [1]. An abrupt narrowing of the LRV with the characteristic “beak sign” is the most significant finding among the CT parameters, with a sensitivity of 91.7% and specificity of 88.9%. MRA findings are like those of CT and are preferred in children because they are less invasive and have lower radiation exposure. Even though it is invasive, venography with direct pressure measurement ±IVUS remains the gold standard for diagnosis as it has both diagnostic and therapeutic value [7].

Treatment options consist of both conservative and surgical methods, with the conservative treatment preferred for patients younger than 18 years and those with mild symptoms. Angiotensin-converting enzyme (ACE) inhibitors are the drugs of choice, as they have shown improvement in long-standing proteinuria in these patients. It is understood that with growth, more fat and fibrous tissue develops, which releases tension on the LRV, thereby improving the symptoms. If conservative therapy fails to relieve symptoms after 24 months of treatment, surgery should be considered. The cut-off in adults is after six months of conservative treatment [3,7].

Multiple surgical options are available, such as LRV transposition, renal autotransplant, and extravascular and intravascular stenting. LRV transposition is the most effective method of treatment and consists of detachment and re-anastomosis of the LRV with the inferior vena cava distal to the superior mesenteric artery, hence removing it from the narrow portion and relieving the tension. This procedure is associated with the risk of LRV thrombosis, with some patients requiring nephrectomy due to intractable hematuria [3,7,8].

Minimally invasive procedures have been adopted for NCS, as in this case. Although there is no dedicated stent for the renal vein, current devices, such as wall-stent (Boston Scientific, Marlborough, MA, USA) and SMART Control (Cordis, Santa Clara CA, USA), have been proven to be effective. The main advantages are the short recovery time and low risk associated with the procedure. The complications associated with the procedure include stent migration and possibility of restenosis and thrombosis [9,10].

## Conclusions

This case illustrates the need of a diagnostic criteria for NCS. It should be considered in patients with recurrent venous congestion symptoms because timely diagnosis and intervention can prevent a host of complications. It also signifies the connectivity of all the venous congestion syndromes and the possibility of MTS progressing to cause bilateral common iliac vein compression.
